# (2*R*,3*S*)-2-Benzyl-3-(2,3,4,6-tetra-*O*-acetyl-β-d-glucopyranos­yloxy)butanolide

**DOI:** 10.1107/S1600536810006628

**Published:** 2010-02-27

**Authors:** Feng Zhang, Jialiang Zhong, Bei Han, Dali Yin, Haihong Huang

**Affiliations:** a Institute of Materia Medica, Chinese Academy of Medical Sciences and Peking Union Medical College, Beijing 100050, People’s Republic of China; b Shanghai Institute of Pharmaceutical Industry, Shanghai 200040, People’s Republic of China

## Abstract

The title compound, C_25_H_30_O_12_, which demonstrates a significant hepatoprotective effect, has comparable geometrical parameters to those of similar compounds. The absolute configuration of the title compound, *viz*. 2*R*,3*S*, was identified from the Flack parameter of 0.05 (17) and the Hooft parameter of 0.04 (6).

## Related literature

For the hepatoprotective effect of the title compound, see: Du & Irinon (2008 ▶). For bond-length data, see: Allen *et al.* (1987 ▶). For the Hooft parameter, see: Hooft *et al.*(2008 ▶). For details of the preparation, see: Saito *et al.* (1992 ▶); Kazumasa *et al.* (2000 ▶); Schmidt (1986 ▶); Corey & Venkateswarlu (1972 ▶); Fernandez *et al.* (1997 ▶).
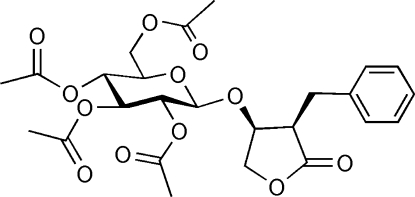


## Experimental

### 

#### Crystal data

C_25_H_30_O_12_
*M* *_r_* = 522.49
Orthorhombic, 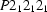

*a* = 10.6142 (2) Å
*b* = 11.0984 (2) Å
*c* = 22.9714 (3) Å
*V* = 2706.05 (8) Å^3^
*Z* = 4
Cu *K*α radiation
μ = 0.87 mm^−1^
*T* = 153 K
0.15 × 0.15 × 0.10 mm

#### Data collection

Mac dip 2030b diffractometer
Absorption correction: multi-scan (*SADABS*; Sheldrick, 2003 ▶) *T*_min_ = 0.880, *T*_max_ = 0.918
7246 measured reflections
4102 independent reflections
4015 reflections with *I* > 2σ(*I*)
*R*_int_ = 0.016

#### Refinement

*R*[*F* ^2^ > 2σ(*F* ^2^)] = 0.036
*wR*(*F* ^2^) = 0.102
*S* = 1.07
4102 reflections
334 parameters
H-atom parameters constrained
Δρ_max_ = 0.34 e Å^−3^
Δρ_min_ = −0.22 e Å^−3^
Absolute structure: Flack (1983 ▶), 1417 Friedel pairs
Flack parameter: 0.05 (17)

### 

Data collection: *DENZO* (Otwinowski & Minor, 1997 ▶); cell refinement: *SCALEPACK* (Otwinowski & Minor, 1997 ▶); data reduction: *SCALEPACK*; program(s) used to solve structure: *SHELXS97* (Sheldrick, 2008 ▶); program(s) used to refine structure: *SHELXL97* (Sheldrick, 2008 ▶); molecular graphics: *ORTEPII* (Johnson, 1976 ▶) and *PLATON* (Spek, 2009 ▶); software used to prepare material for publication: *SHELXL97*.

## Supplementary Material

Crystal structure: contains datablocks global, I. DOI: 10.1107/S1600536810006628/vm2019sup1.cif

Structure factors: contains datablocks I. DOI: 10.1107/S1600536810006628/vm2019Isup2.hkl

Additional supplementary materials:  crystallographic information; 3D view; checkCIF report

## Figures and Tables

**Fig. 1.**
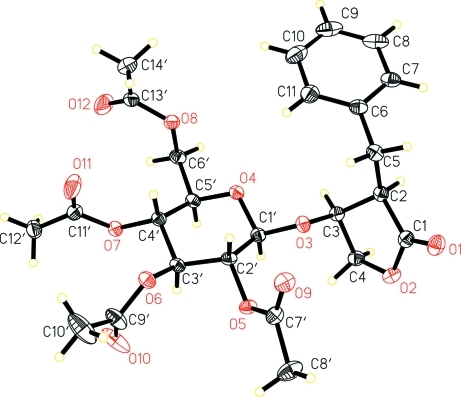
View of the title compound showing the atom-labelling scheme. Displacement ellipsoids are drawn at the 30% probability level.

## supplementary crystallographic information

### Comment

The title compound was found to have a significant hepatoprotective effect, comparable to the natural product Goodyeroside A (Du & Irinon, 2008). Recently, the compound was successfully crystallized from a petroleum ether/EtOAc mixture, yielding crystals suitable for X-ray analysis.

Fig.1 shows the molecular structure of the title compound with atomic numbering scheme. The bond lengths and angles are in agreement with reported literature values (Table 1) (Allen *et al.*, 1987). The benzene ring (C6—C11) is essentially planar, with r.m.s deviations of 0.0078 (14) Å. The five-membered ring (C1—C4/O2) has an envelope conformation with the C3 atom out of plane and the six-membered ring (C1'-C5'/O4) is in its chair conformation. The dihedral angles between the various rings in the title compound are as follows, where the first atom is used to identify its five- or six- membered ring: C1/C6 112.9 (1)°; C1/C1' 110.1 (1)°; C6/C1' 13.1 (1)°.

In order to determine the absolute configuration of the title compound, the data collection was performed using Cu Kα. The absolute configuration is confirmed by the Flack parameter 0.05 (17) and Hooft parameter 0.04 (6) (Flack, 1983; Hooft *et al.*, 2008).

### Experimental

To a solution of Ethyl (2*R*,3*S*)-3-(2,3,4,6-tetra-*O*-acetyl-β-D-glucopyranosyloxy)-2- benzyl-4-hydroxybutanoate (1.0 equiv) in 1,4-dioxane (10 ml) was added 4-methylbenzenesulfonic acid (1.0 equiv), then the solution was stirred at room temperature for 2 days. After removal of the solvent in vacuo, the residue was purified by column chromatography. White crystals suitable for X-ray analysis were obtained by slow evaportion of a petroleum ether/EtOAc solution over a period of two weeks (Saito *et al.*, 1992; Kazumasa *et al.*, 2000; Schmidt, 1986; Corey & Venkateswarlu, 1972; Fernandez *et al.*, 1997).

### Refinement

All H atoms were placed in geometrically idealised positions and constrained to ride on their parent atoms with C—H distances in the range of 0.95-1.00 Å, with a displacement parameter *U*_iso_ set to 1.2 (CH and CH_2_) or 1.5(CH_3_) times *U*_eq_ of the parent atom.

### Crystal data

**Table:**

C_25_H_30_O_12_	*F*(000) = 1104
*M**_r_* = 522.49	*D*_x_ = 1.282 Mg m^−^^3^
Orthorhombic, *P*2_1_2_1_2_1_	Cu *K*α radiation, λ = 1.54178 Å
Hall symbol: P 2ac 2ab	Cell parameters from 5304 reflections
*a* = 10.6142 (2) Å	θ = 4.0–65.7°
*b* = 11.0984 (2) Å	µ = 0.87 mm^−^^1^
*c* = 22.9714 (3) Å	*T* = 153 K
*V* = 2706.05 (8) Å^3^	Block, white
*Z* = 4	0.15 × 0.15 × 0.10 mm

### Data collection

**Table:**

Mac dip 2030b diffractometer	4102 independent reflections
Radiation source: rotating anode	4015 reflections with *I* > 2σ(*I*)
graphite	*R*_int_ = 0.016
Detector resolution: 0 pixels mm^-1^	θ_max_ = 66.0°, θ_min_ = 4.4°
ω scans	*h* = −12→9
Absorption correction: multi-scan (*SADABS*; Sheldrick, 2003)	*k* = −12→12
*T*_min_ = 0.880, *T*_max_ = 0.918	*l* = −26→26
7246 measured reflections	

### Refinement

**Table:**

Refinement on *F*^2^	Secondary atom site location: difference Fourier map
Least-squares matrix: full	Hydrogen site location: inferred from neighbouring sites
*R*[*F*^2^ > 2σ(*F*^2^)] = 0.036	H-atom parameters constrained
*wR*(*F*^2^) = 0.102	*w* = 1/[σ^2^(*F*_o_^2^) + (0.0654*P*)^2^ + 0.5803*P*] where *P* = (*F*_o_^2^ + 2*F*_c_^2^)/3
*S* = 1.07	(Δ/σ)_max_ < 0.001
4102 reflections	Δρ_max_ = 0.34 e Å^−^^3^
334 parameters	Δρ_min_ = −0.22 e Å^−^^3^
0 restraints	Absolute structure: Flack (1983), 1417 Friedel pairs
Primary atom site location: structure-invariant direct methods	Flack parameter: 0.05 (17)

### Special details

**Table:**

Geometry. All esds (except the esd in the dihedral angle between two l.s. planes) are estimated using the full covariance matrix. The cell esds are taken into account individually in the estimation of esds in distances, angles and torsion angles; correlations between esds in cell parameters are only used when they are defined by crystal symmetry. An approximate (isotropic) treatment of cell esds is used for estimating esds involving l.s. planes.
Refinement. Refinement of *F*^2^ against ALL reflections. The weighted *R*-factor wR and goodness of fit *S* are based on *F*^2^, conventional *R*-factors *R* are based on *F*, with *F* set to zero for negative *F*^2^. The threshold expression of *F*^2^ > σ(*F*^2^) is used only for calculating *R*-factors (gt) etc. and is not relevant to the choice of reflections for refinement. *R*-factors based on *F*^2^ are statistically about twice as large as those based on *F*, and *R*- factors based on ALL data will be even larger.

### Fractional atomic coordinates and isotropic or equivalent isotropic displacement parameters (Å^2^)

**Table:**

	*x*	*y*	*z*	*U*_iso_*/*U*_eq_	
O1	0.47650 (16)	0.31851 (14)	0.32119 (7)	0.0442 (4)	
O2	0.58032 (14)	0.48590 (14)	0.29725 (6)	0.0403 (3)	
O3	0.49559 (12)	0.47669 (12)	0.17568 (6)	0.0311 (3)	
O4	0.44988 (12)	0.58270 (12)	0.09414 (6)	0.0300 (3)	
O5	0.73101 (12)	0.40529 (12)	0.12561 (6)	0.0319 (3)	
O6	0.71425 (13)	0.39829 (13)	0.00188 (6)	0.0351 (3)	
O7	0.61363 (13)	0.62268 (12)	−0.04400 (6)	0.0325 (3)	
O8	0.29897 (12)	0.61966 (13)	−0.00230 (6)	0.0353 (3)	
O9	0.64011 (15)	0.22826 (13)	0.14807 (7)	0.0427 (4)	
O10	0.89389 (19)	0.49770 (18)	−0.01722 (12)	0.0784 (7)	
O11	0.5236 (2)	0.4937 (2)	−0.10623 (7)	0.0675 (6)	
O12	0.2865 (2)	0.7043 (2)	−0.09013 (8)	0.0739 (7)	
C1	0.4778 (2)	0.41371 (19)	0.29662 (9)	0.0341 (4)	
C2	0.37455 (19)	0.47400 (18)	0.26180 (8)	0.0306 (4)	
H2A	0.3281	0.5283	0.2891	0.037*	
C3	0.45018 (18)	0.55428 (17)	0.22133 (8)	0.0297 (4)	
H3A	0.4010	0.6247	0.2064	0.036*	
C4	0.5581 (2)	0.59050 (19)	0.26080 (9)	0.0351 (4)	
H4A	0.6341	0.6103	0.2377	0.042*	
H4B	0.5350	0.6613	0.2847	0.042*	
C5	0.2771 (2)	0.38840 (18)	0.23518 (9)	0.0346 (4)	
H5A	0.3174	0.3409	0.2038	0.042*	
H5B	0.2477	0.3314	0.2654	0.042*	
C6	0.16563 (19)	0.45524 (18)	0.21066 (10)	0.0354 (4)	
C7	0.0715 (2)	0.4974 (2)	0.24720 (10)	0.0421 (5)	
H7A	0.0756	0.4808	0.2877	0.050*	
C8	−0.0285 (2)	0.5636 (2)	0.22519 (15)	0.0569 (7)	
H8A	−0.0916	0.5928	0.2509	0.068*	
C9	−0.0377 (3)	0.5875 (2)	0.16642 (15)	0.0616 (8)	
H9A	−0.1064	0.6328	0.1515	0.074*	
C10	0.0552 (3)	0.5443 (3)	0.12938 (13)	0.0606 (8)	
H10A	0.0493	0.5591	0.0887	0.073*	
C11	0.1569 (2)	0.4795 (2)	0.15118 (10)	0.0456 (5)	
H11A	0.2207	0.4516	0.1255	0.055*	
C1'	0.54911 (18)	0.53583 (18)	0.12866 (8)	0.0294 (4)	
H1'A	0.6062	0.6018	0.1421	0.035*	
C2'	0.62123 (18)	0.44333 (18)	0.09365 (8)	0.0283 (4)	
H2'A	0.5661	0.3723	0.0854	0.034*	
C3'	0.66843 (17)	0.49655 (17)	0.03700 (8)	0.0281 (4)	
H3'A	0.7380	0.5551	0.0448	0.034*	
C4'	0.56170 (18)	0.55880 (17)	0.00501 (8)	0.0288 (4)	
H4'A	0.4997	0.4974	−0.0089	0.035*	
C5'	0.49602 (18)	0.64833 (18)	0.04534 (8)	0.0299 (4)	
H5'A	0.5582	0.7100	0.0589	0.036*	
C6'	0.38565 (19)	0.71066 (19)	0.01696 (9)	0.0359 (5)	
H6'A	0.3439	0.7650	0.0452	0.043*	
H6'B	0.4148	0.7593	−0.0166	0.043*	
C7'	0.7291 (2)	0.29459 (19)	0.15016 (9)	0.0351 (4)	
C8'	0.8527 (3)	0.2666 (2)	0.17810 (13)	0.0547 (6)	
H8'A	0.8493	0.1860	0.1955	0.082*	
H8'B	0.8703	0.3262	0.2085	0.082*	
H8'C	0.9195	0.2692	0.1487	0.082*	
C9'	0.8281 (2)	0.4122 (2)	−0.02386 (12)	0.0516 (6)	
C10'	0.8550 (4)	0.3043 (3)	−0.06145 (19)	0.0899 (12)	
H10B	0.9370	0.3143	−0.0804	0.135*	
H10C	0.7892	0.2967	−0.0912	0.135*	
H10D	0.8562	0.2315	−0.0373	0.135*	
C11'	0.5904 (2)	0.5778 (2)	−0.09765 (9)	0.0385 (5)	
C12'	0.6610 (2)	0.6479 (2)	−0.14304 (10)	0.0464 (5)	
H12A	0.6431	0.6142	−0.1816	0.070*	
H12B	0.7516	0.6428	−0.1353	0.070*	
H12C	0.6343	0.7324	−0.1419	0.070*	
C13'	0.26186 (19)	0.6221 (2)	−0.05781 (9)	0.0403 (5)	
C14'	0.1842 (2)	0.5152 (3)	−0.07186 (11)	0.0526 (6)	
H14A	0.1585	0.5186	−0.1128	0.079*	
H14B	0.1091	0.5144	−0.0470	0.079*	
H14C	0.2334	0.4419	−0.0650	0.079*	

### Atomic displacement parameters (Å^2^)

**Table:**

	*U*^11^	*U*^22^	*U*^33^	*U*^12^	*U*^13^	*U*^23^
O1	0.0555 (9)	0.0361 (8)	0.0410 (8)	0.0107 (7)	−0.0020 (7)	0.0053 (7)
O2	0.0368 (8)	0.0424 (9)	0.0418 (7)	0.0029 (7)	−0.0093 (6)	−0.0006 (7)
O3	0.0359 (7)	0.0277 (7)	0.0297 (6)	−0.0020 (5)	0.0038 (5)	−0.0030 (6)
O4	0.0273 (7)	0.0310 (7)	0.0318 (6)	0.0005 (6)	0.0018 (5)	−0.0016 (6)
O5	0.0276 (7)	0.0281 (7)	0.0401 (7)	−0.0025 (6)	−0.0058 (6)	0.0042 (6)
O6	0.0340 (7)	0.0320 (7)	0.0393 (7)	0.0047 (6)	0.0059 (6)	−0.0049 (6)
O7	0.0321 (7)	0.0342 (7)	0.0313 (6)	−0.0013 (6)	0.0026 (6)	0.0017 (6)
O8	0.0294 (7)	0.0396 (8)	0.0369 (7)	−0.0006 (6)	−0.0016 (6)	0.0059 (6)
O9	0.0455 (9)	0.0308 (8)	0.0518 (8)	−0.0081 (7)	−0.0055 (7)	0.0073 (7)
O10	0.0542 (11)	0.0501 (12)	0.1310 (19)	−0.0041 (10)	0.0518 (12)	−0.0067 (12)
O11	0.0873 (14)	0.0811 (15)	0.0342 (8)	−0.0362 (13)	−0.0059 (8)	−0.0060 (9)
O12	0.0730 (13)	0.0984 (17)	0.0504 (10)	−0.0315 (12)	−0.0147 (9)	0.0310 (11)
C1	0.0384 (11)	0.0333 (11)	0.0305 (9)	0.0050 (9)	0.0005 (8)	−0.0050 (9)
C2	0.0327 (10)	0.0287 (10)	0.0303 (9)	0.0043 (8)	0.0020 (8)	−0.0028 (8)
C3	0.0300 (10)	0.0271 (10)	0.0320 (9)	0.0022 (8)	−0.0005 (8)	−0.0042 (8)
C4	0.0331 (10)	0.0339 (11)	0.0383 (10)	−0.0011 (8)	−0.0029 (8)	−0.0039 (9)
C5	0.0345 (10)	0.0280 (10)	0.0413 (10)	−0.0006 (9)	0.0030 (9)	−0.0007 (9)
C6	0.0317 (10)	0.0280 (10)	0.0464 (11)	−0.0061 (8)	−0.0026 (9)	−0.0024 (9)
C7	0.0312 (10)	0.0416 (12)	0.0534 (12)	−0.0050 (9)	−0.0025 (9)	−0.0091 (11)
C8	0.0311 (12)	0.0459 (14)	0.094 (2)	−0.0005 (10)	−0.0113 (12)	−0.0126 (14)
C9	0.0429 (14)	0.0391 (13)	0.103 (2)	−0.0070 (11)	−0.0301 (15)	0.0106 (15)
C10	0.0598 (17)	0.0572 (16)	0.0650 (16)	−0.0182 (14)	−0.0268 (14)	0.0211 (14)
C11	0.0473 (12)	0.0424 (13)	0.0471 (12)	−0.0111 (11)	−0.0049 (10)	0.0031 (10)
C1'	0.0280 (9)	0.0284 (10)	0.0317 (9)	−0.0053 (8)	−0.0001 (8)	−0.0017 (8)
C2'	0.0234 (9)	0.0271 (10)	0.0342 (9)	−0.0028 (7)	−0.0031 (8)	0.0009 (8)
C3'	0.0251 (9)	0.0255 (9)	0.0337 (9)	−0.0003 (7)	0.0034 (7)	−0.0034 (8)
C4'	0.0273 (9)	0.0280 (10)	0.0310 (9)	−0.0028 (7)	0.0018 (8)	−0.0006 (8)
C5'	0.0277 (10)	0.0282 (10)	0.0338 (9)	−0.0011 (7)	0.0027 (8)	−0.0001 (8)
C6'	0.0309 (10)	0.0329 (11)	0.0439 (11)	0.0045 (9)	0.0021 (9)	0.0018 (9)
C7'	0.0404 (11)	0.0284 (10)	0.0366 (10)	0.0008 (9)	−0.0039 (9)	0.0014 (8)
C8'	0.0558 (15)	0.0383 (13)	0.0701 (15)	−0.0020 (11)	−0.0260 (13)	0.0120 (12)
C9'	0.0476 (13)	0.0386 (13)	0.0686 (15)	0.0099 (11)	0.0254 (12)	0.0046 (12)
C10'	0.087 (2)	0.0606 (18)	0.122 (3)	0.0138 (17)	0.060 (2)	−0.014 (2)
C11'	0.0371 (12)	0.0449 (13)	0.0335 (10)	0.0044 (10)	−0.0032 (9)	−0.0009 (9)
C12'	0.0471 (13)	0.0555 (14)	0.0365 (10)	0.0082 (11)	0.0073 (10)	0.0059 (10)
C13'	0.0272 (10)	0.0571 (14)	0.0367 (10)	0.0039 (9)	−0.0007 (9)	0.0101 (10)
C14'	0.0434 (13)	0.0680 (17)	0.0464 (12)	−0.0034 (12)	−0.0112 (10)	0.0012 (12)

### Geometric parameters (Å, °)

**Table:**

O1—C1	1.198 (3)	C8—H8A	0.9500
O2—C1	1.351 (3)	C9—C10	1.388 (5)
O2—C4	1.451 (3)	C9—H9A	0.9500
O3—C1'	1.386 (2)	C10—C11	1.390 (4)
O3—C3	1.440 (2)	C10—H10A	0.9500
O4—C1'	1.417 (2)	C11—H11A	0.9500
O4—C5'	1.424 (2)	C1'—C2'	1.512 (3)
O5—C7'	1.352 (3)	C1'—H1'A	1.0000
O5—C2'	1.440 (2)	C2'—C3'	1.514 (3)
O6—C9'	1.355 (3)	C2'—H2'A	1.0000
O6—C3'	1.441 (2)	C3'—C4'	1.517 (3)
O7—C11'	1.352 (2)	C3'—H3'A	1.0000
O7—C4'	1.440 (2)	C4'—C5'	1.527 (3)
O8—C13'	1.335 (3)	C4'—H4'A	1.0000
O8—C6'	1.436 (3)	C5'—C6'	1.509 (3)
O9—C7'	1.199 (3)	C5'—H5'A	1.0000
O10—C9'	1.188 (3)	C6'—H6'A	0.9900
O11—C11'	1.189 (3)	C6'—H6'B	0.9900
O12—C13'	1.205 (3)	C7'—C8'	1.493 (3)
C1—C2	1.513 (3)	C8'—H8'A	0.9800
C2—C3	1.517 (3)	C8'—H8'B	0.9800
C2—C5	1.532 (3)	C8'—H8'C	0.9800
C2—H2A	1.0000	C9'—C10'	1.504 (4)
C3—C4	1.515 (3)	C10'—H10B	0.9800
C3—H3A	1.0000	C10'—H10C	0.9800
C4—H4A	0.9900	C10'—H10D	0.9800
C4—H4B	0.9900	C11'—C12'	1.501 (3)
C5—C6	1.506 (3)	C12'—H12A	0.9800
C5—H5A	0.9900	C12'—H12B	0.9800
C5—H5B	0.9900	C12'—H12C	0.9800
C6—C7	1.386 (3)	C13'—C14'	1.480 (4)
C6—C11	1.396 (3)	C14'—H14A	0.9800
C7—C8	1.387 (3)	C14'—H14B	0.9800
C7—H7A	0.9500	C14'—H14C	0.9800
C8—C9	1.379 (5)		
			
C1—O2—C4	109.73 (15)	C1'—C2'—H2'A	109.8
C1'—O3—C3	114.93 (15)	C3'—C2'—H2'A	109.8
C1'—O4—C5'	111.88 (14)	O6—C3'—C2'	107.32 (15)
C7'—O5—C2'	117.84 (15)	O6—C3'—C4'	108.99 (15)
C9'—O6—C3'	117.33 (17)	C2'—C3'—C4'	110.30 (15)
C11'—O7—C4'	117.49 (16)	O6—C3'—H3'A	110.1
C13'—O8—C6'	117.98 (17)	C2'—C3'—H3'A	110.1
O1—C1—O2	121.81 (19)	C4'—C3'—H3'A	110.1
O1—C1—C2	129.1 (2)	O7—C4'—C3'	108.49 (15)
O2—C1—C2	109.08 (17)	O7—C4'—C5'	109.19 (15)
C1—C2—C3	101.56 (16)	C3'—C4'—C5'	110.08 (15)
C1—C2—C5	115.21 (17)	O7—C4'—H4'A	109.7
C3—C2—C5	118.49 (16)	C3'—C4'—H4'A	109.7
C1—C2—H2A	106.9	C5'—C4'—H4'A	109.7
C3—C2—H2A	106.9	O4—C5'—C6'	107.93 (16)
C5—C2—H2A	106.9	O4—C5'—C4'	107.57 (15)
O3—C3—C4	109.95 (16)	C6'—C5'—C4'	112.99 (16)
O3—C3—C2	105.78 (15)	O4—C5'—H5'A	109.4
C4—C3—C2	100.91 (16)	C6'—C5'—H5'A	109.4
O3—C3—H3A	113.1	C4'—C5'—H5'A	109.4
C4—C3—H3A	113.1	O8—C6'—C5'	107.95 (16)
C2—C3—H3A	113.1	O8—C6'—H6'A	110.1
O2—C4—C3	104.82 (16)	C5'—C6'—H6'A	110.1
O2—C4—H4A	110.8	O8—C6'—H6'B	110.1
C3—C4—H4A	110.8	C5'—C6'—H6'B	110.1
O2—C4—H4B	110.8	H6'A—C6'—H6'B	108.4
C3—C4—H4B	110.8	O9—C7'—O5	123.58 (19)
H4A—C4—H4B	108.9	O9—C7'—C8'	125.6 (2)
C6—C5—C2	111.99 (16)	O5—C7'—C8'	110.83 (18)
C6—C5—H5A	109.2	C7'—C8'—H8'A	109.5
C2—C5—H5A	109.2	C7'—C8'—H8'B	109.5
C6—C5—H5B	109.2	H8'A—C8'—H8'B	109.5
C2—C5—H5B	109.2	C7'—C8'—H8'C	109.5
H5A—C5—H5B	107.9	H8'A—C8'—H8'C	109.5
C7—C6—C11	118.7 (2)	H8'B—C8'—H8'C	109.5
C7—C6—C5	120.4 (2)	O10—C9'—O6	124.0 (2)
C11—C6—C5	120.9 (2)	O10—C9'—C10'	126.8 (2)
C8—C7—C6	120.6 (2)	O6—C9'—C10'	109.2 (2)
C8—C7—H7A	119.7	C9'—C10'—H10B	109.5
C6—C7—H7A	119.7	C9'—C10'—H10C	109.5
C9—C8—C7	120.9 (3)	H10B—C10'—H10C	109.5
C9—C8—H8A	119.6	C9'—C10'—H10D	109.5
C7—C8—H8A	119.6	H10B—C10'—H10D	109.5
C8—C9—C10	118.9 (2)	H10C—C10'—H10D	109.5
C8—C9—H9A	120.5	O11—C11'—O7	123.3 (2)
C10—C9—H9A	120.5	O11—C11'—C12'	126.1 (2)
C9—C10—C11	120.6 (3)	O7—C11'—C12'	110.57 (19)
C9—C10—H10A	119.7	C11'—C12'—H12A	109.5
C11—C10—H10A	119.7	C11'—C12'—H12B	109.5
C10—C11—C6	120.3 (3)	H12A—C12'—H12B	109.5
C10—C11—H11A	119.9	C11'—C12'—H12C	109.5
C6—C11—H11A	119.9	H12A—C12'—H12C	109.5
O3—C1'—O4	107.78 (15)	H12B—C12'—H12C	109.5
O3—C1'—C2'	107.49 (16)	O12—C13'—O8	122.7 (2)
O4—C1'—C2'	109.14 (14)	O12—C13'—C14'	126.4 (2)
O3—C1'—H1'A	110.8	O8—C13'—C14'	110.89 (19)
O4—C1'—H1'A	110.8	C13'—C14'—H14A	109.5
C2'—C1'—H1'A	110.8	C13'—C14'—H14B	109.5
O5—C2'—C1'	109.72 (15)	H14A—C14'—H14B	109.5
O5—C2'—C3'	106.54 (15)	C13'—C14'—H14C	109.5
C1'—C2'—C3'	111.08 (16)	H14A—C14'—H14C	109.5
O5—C2'—H2'A	109.8	H14B—C14'—H14C	109.5
			
C4—O2—C1—O1	176.02 (19)	O3—C1'—C2'—O5	−70.08 (18)
C4—O2—C1—C2	−4.4 (2)	O4—C1'—C2'—O5	173.29 (14)
O1—C1—C2—C3	−154.5 (2)	O3—C1'—C2'—C3'	172.38 (15)
O2—C1—C2—C3	25.89 (19)	O4—C1'—C2'—C3'	55.8 (2)
O1—C1—C2—C5	−25.2 (3)	C9'—O6—C3'—C2'	−132.97 (19)
O2—C1—C2—C5	155.26 (16)	C9'—O6—C3'—C4'	107.6 (2)
C1'—O3—C3—C4	−82.37 (19)	O5—C2'—C3'—O6	71.25 (18)
C1'—O3—C3—C2	169.45 (14)	C1'—C2'—C3'—O6	−169.29 (14)
C1—C2—C3—O3	79.21 (17)	O5—C2'—C3'—C4'	−170.14 (15)
C5—C2—C3—O3	−48.1 (2)	C1'—C2'—C3'—C4'	−50.7 (2)
C1—C2—C3—C4	−35.34 (18)	C11'—O7—C4'—C3'	108.25 (18)
C5—C2—C3—C4	−162.61 (17)	C11'—O7—C4'—C5'	−131.76 (18)
C1—O2—C4—C3	−19.3 (2)	O6—C3'—C4'—O7	−70.62 (18)
O3—C3—C4—O2	−77.30 (19)	C2'—C3'—C4'—O7	171.80 (15)
C2—C3—C4—O2	34.09 (19)	O6—C3'—C4'—C5'	169.95 (14)
C1—C2—C5—C6	169.70 (17)	C2'—C3'—C4'—C5'	52.4 (2)
C3—C2—C5—C6	−69.8 (2)	C1'—O4—C5'—C6'	−171.17 (16)
C2—C5—C6—C7	−78.9 (2)	C1'—O4—C5'—C4'	66.61 (18)
C2—C5—C6—C11	98.9 (2)	O7—C4'—C5'—O4	−177.99 (14)
C11—C6—C7—C8	−0.8 (3)	C3'—C4'—C5'—O4	−58.99 (19)
C5—C6—C7—C8	177.1 (2)	O7—C4'—C5'—C6'	63.0 (2)
C6—C7—C8—C9	0.9 (4)	C3'—C4'—C5'—C6'	−178.01 (16)
C7—C8—C9—C10	−0.1 (4)	C13'—O8—C6'—C5'	−127.09 (18)
C8—C9—C10—C11	−1.0 (4)	O4—C5'—C6'—O8	−61.5 (2)
C9—C10—C11—C6	1.1 (4)	C4'—C5'—C6'—O8	57.4 (2)
C7—C6—C11—C10	−0.3 (3)	C2'—O5—C7'—O9	−2.1 (3)
C5—C6—C11—C10	−178.1 (2)	C2'—O5—C7'—C8'	176.30 (19)
C3—O3—C1'—O4	−77.32 (18)	C3'—O6—C9'—O10	3.2 (4)
C3—O3—C1'—C2'	165.16 (15)	C3'—O6—C9'—C10'	−176.3 (2)
C5'—O4—C1'—O3	178.36 (14)	C4'—O7—C11'—O11	4.8 (3)
C5'—O4—C1'—C2'	−65.20 (19)	C4'—O7—C11'—C12'	−174.66 (18)
C7'—O5—C2'—C1'	106.05 (19)	C6'—O8—C13'—O12	−8.6 (3)
C7'—O5—C2'—C3'	−133.61 (17)	C6'—O8—C13'—C14'	173.88 (18)
